# 206 Purpose and Audience of Public Policies Promoting Physical Activity and Reducing Sedentary Behavior in South American Countries

**DOI:** 10.1093/eurpub/ckae114.105

**Published:** 2024-09-26

**Authors:** Ingrid Kelly Alves Dos Santos Pinheiro, Gerson Luis de Moraes Ferrari, Danilo Rodrigues Pereira da Silva

**Affiliations:** Postgraduate Program in Health Sciences, Federal University of Sergipe, Aracaju, Brazil; University of Santiago de Chile, Santiago, Chile; Postgraduate Program in Health Sciences, Federal University of Sergipe, Aracaju, Brazil

## Abstract

**Objective:**

To explore the purpose and audience of public policies for promoting physical activity and reducing sedentary behavior in South American countries.

**Methods:**

This is a descriptive and documentary research conducted in five steps: 1) identification of representatives in South American countries, including key researchers, professionals, and/or policymakers in the field; 2) search for policies already cataloged in the inventory of the Global Observatory for Physical Activity; 3) consultation with national representatives of the South American Physical Activity and Sedentary Behavior Network regarding the main public policies and contacts of individuals directly and/or indirectly involved in these policies in their countries; 4) search for policies on official websites in each of the countries. Documents presenting details on physical activity policy, program, or national plan were included, representing only national-level policies. Following the Comprehensive Analysis of Policy on Physical Activity (CAPPA), information from the identified documents was extracted into a Microsoft Excel spreadsheet and presented by country/document, with a descriptive analysis of the data.

**Results:**

55 public policies for promoting physical activity were identified, with 25% found in Chile, and no documents regarding the reduction of sedentary behavior. Most documents (42%) focused exclusively on promoting physical activity. Furthermore, these documents target the general population (82%); however, there are no documents specifically targeting women, pregnant women, elderly individuals, or people with disabilities.

**Conclusions:**

A significant portion of the documents are public policies aimed at promoting physical activity for the general population. There should be encouragement for the development of public policies that also aim to reduce sedentary behavior, as well as policies that are inclusive of populations that, for various reasons, are more likely to have low levels of physical activity.

